# Patterns and Trends in Cancer Screening in the United States

**DOI:** 10.5888/pcd15.170465

**Published:** 2018-07-26

**Authors:** Ingrid J. Hall, Florence K.L. Tangka, Susan A. Sabatino, Trevor D. Thompson, Barry I. Graubard, Nancy Breen

**Affiliations:** 1 Division of Cancer Prevention and Control, National Center for Chronic Disease Prevention and Health Promotion, Centers for Disease Control and Prevention, Atlanta, Georgia; 2 Division of Cancer Epidemiology and Genetics, National Cancer Institute, Bethesda, Maryland; 3 Office of Science Policy, Strategic Planning, Assessment, Analyses, Resources, Reporting and Data, National Institute on Minority Health and Health Disparities, Bethesda, Maryland

## Abstract

**Introduction:**

We examined the prevalence of cancer screening reported in 2015 among US adults, adjusted for important sociodemographic and access-to-care variables. By using data from the National Health Interview Survey (NHIS) for 2000 through 2015, we examined trends in prevalence of cancer screening that adhered to US Preventive Services Task Force screening recommendations in order to monitor screening progress among traditionally underserved population subgroups.

**Methods:**

We analyzed NHIS data from surveys from 2000 through 2015 to estimate prevalence and trends in use of recommended screening tests for breast, cervical, colorectal, and prostate cancers. We used logistic regression and report predictive margins for population subgroups adjusted for various socioeconomic and demographic variables.

**Results:**

Colorectal cancer screening was the only test that increased during the study period. We found disparities in prevalence of test use among subgroups for all tests examined. Factors that reduced the use of screening tests included no contact with a doctor in the past year, no usual source of health care, and no insurance coverage.

**Conclusion:**

Understanding use of cancer screening tests among different population subgroups is vital for planning public health interventions with potential to increase screening uptake and reduce disparities in cancer morbidity and mortality. Overarching goals of Healthy People 2020 are to “achieve health equity, eliminate disparities, and improve the health of all groups.” Adjusted findings for 2015, compared with previous years, show persistent screening disparities, particularly among the uninsured, and progress for colorectal cancer screening only.

**MEDSCAPE CME**

Medscape, LLC is pleased to provide online continuing medical education (CME) for this journal article, allowing clinicians the opportunity to earn CME credit.

In support of improving patient care, this activity has been planned and implemented by Medscape, LLC and *Preventing Chronic Disease*. Medscape, LLC is jointly accredited by the Accreditation Council for Continuing Medical Education (ACCME), the Accreditation Council for Pharmacy Education (ACPE), and the American Nurses Credentialing Center (ANCC), to provide continuing education for the healthcare team.

Medscape, LLC designates this Journal-based CME activity for a maximum of 1.00 *AMA PRA Category 1 Credit(s)™*. Physicians should claim only the credit commensurate with the extent of their participation in the activity.

All other clinicians completing this activity will be issued a certificate of participation. To participate in this journal CME activity: (1) review the learning objectives and author disclosures; (2) study the education content; (3) take the post-test with a 75% minimum passing score and complete the evaluation at http://www.medscape.org/journal/pcd; (4) view/print certificate.

**Release date: July 26, 2018; Expiration date: July 26, 2019**

**Learning Objectives**

Upon completion of this activity, participants will be able to:

1. Evaluate specific trends in the prevalence of cancer screening, based on an analysis of 2000 to 2015 National Health Interview Survey (NHIS) data
2. Assess factors associated with underuse of cancer screening, based on an analysis of 2000 to 2015 NHIS data
3. Determine the clinical implications of trends in prevalence of cancer screening, based on an analysis of 2000 to 2015 NHIS data

**EDITOR**

Rosemarie Perrin

Editor, *Preventing Chronic Disease*

Disclosure: Rosemarie Perrin has disclosed no relevant financial relationships.

**CME AUTHOR**

Laurie Barclay, MD

Freelance writer and reviewer, Medscape, LLC

Disclosure: Laurie Barclay, MD, has disclosed the following relevant financial relationships:

Owns stock, stock options, or bonds from: Pfizer

**AUTHORS**

Ingrid J. Hall, PhD, MPH

Division of Cancer Prevention and Control, National Center for Chronic Disease Prevention and Health Promotion, Centers for Disease Control and Prevention, Atlanta, Georgia

Disclosure: Ingrid J. Hall, PhD, MPH, has disclosed no relevant financial relationships. 

Florence K.L. Tangka, PhD

Division of Cancer Prevention and Control, National Center for Chronic Disease Prevention and Health Promotion, Centers for Disease Control and Prevention, Atlanta, Georgia

Disclosure: Florence K.L. Tangka, PhD, has disclosed no relevant financial relationships. 

Susan A. Sabatino, MD, MPH

Division of Cancer Prevention and Control, National Center for Chronic Disease Prevention and Health Promotion, Centers for Disease Control and Prevention, Atlanta, Georgia

Disclosure: Susan A. Sabatino, MD, MPH, has disclosed the following relevant financial relationships:

Owns stock, stock options, or bonds from: Pfizer 

Trevor D. Thompson, BS

Division of Cancer Prevention and Control, National Center for Chronic Disease Prevention and Health Promotion, Centers for Disease Control and Prevention, Atlanta, Georgia

Disclosure: Trevor D. Thompson, BS, has disclosed no relevant financial relationships. 

Barry I. Graubard, PhD

Division of Cancer Prevention and Population Sciences, National Cancer Institute, Bethesda, Maryland

Disclosure: Barry I. Graubard, PhD, has disclosed no relevant financial relationships. 

Nancy Breen, PhD

Division of Cancer Prevention and Population Sciences, National Cancer Institute, Bethesda, Maryland; Office of Science Policy, Strategic Planning, Assessment, Analyses, Resources, Reporting and Data, National Institute on Minority Health and Health Disparities, Bethesda, Maryland

Disclosure: Nancy Breen, PhD, has disclosed no relevant financial relationships. 

## Introduction

Breast, cervical, colorectal, and prostate cancers cause significant health burdens in the United States. In 2013, these cancers accounted for nearly 40% of all new cancer diagnoses and about 20% of cancer deaths (1). Screening is a primary tool for early detection and reduction in deaths from cancer. Examining screening disparities — differences in receipt of screening among population subgroups — over time offers the opportunity to monitor cancer screening successes and advancement toward Healthy People 2020 goals (2).

Healthy People 2020 objectives for use of cancer screening tests include increasing the proportion of women aged 21 to 65 screened for cervical cancer, women aged 50 to 74 screened for breast cancer, and men and women aged 50 to 75 screened for colorectal cancer (2). Healthy People 2020 objectives also include reducing prostate cancer deaths (2). Routine prostate-specific antigen (PSA) testing was not recommended (3) at the time of this analysis; however, test use was common (4).

The National Health Interview Survey (NHIS) has been used for more than 30 years to measure the nation’s progress toward meeting Healthy People objectives for cancer screening (2). This article examines the sex-specific prevalence of use of screening tests for cervical, breast, colorectal, and prostate cancers reported by adults in NHIS 2015. Adjusting for sociodemographic characteristics and access to care factors, we assessed differences in screening among underserved groups. We examined screening trends from NHIS for 2000, 2003, 2005, 2008, 2010, 2013, and 2015, testing for possible interactions to assess whether use of cancer screening tests in the United States changed overall and for subgroups over those time periods. We assessed progress toward reducing screening disparities, which should aid in decreasing the overall burden of cancer in the United States. This information can be used to assess the persistent unmet needs of underserved groups and to set priorities for interventions to reduce disparities.

## Methods

### National Health Interview Survey

NHIS (www.cdc.gov/nchs/nhis/index.htm), a nationally representative cross-sectional sample of the civilian, noninstitutionalized, US population, is the principal source of information on the health of the nation. The survey excludes residents of long-term care facilities, people on active duty with the Armed Forces (their dependents are included), people incarcerated in the prison system, and US citizens living in foreign countries. NHIS collects sociodemographic and health information via in-person interviews for each participating household. Interviews are conducted by US Census Bureau. The NHIS annual questionnaire includes periodic supplements that cover one or more sets of questions on specific health topics, including cancer screening. 

NHIS Cancer Control Supplements were fielded in 2000, 2003, 2005, 2008, 2010, 2013, and 2015. The final sample adult response rates ranged from 55.2% (2015) to 74.2% (2003) (5). Respondents were asked questions regarding Papanicolaou (Pap) tests and hysterectomy, mammograms, PSA tests, and endoscopic exams and fecal occult blood tests (FOBT) screening for colorectal cancer. For each type of cancer screening, respondents who reported having a test were asked when they had the most recent test.

We used NHIS 2015 data to estimate prevalence of cancer screening. Our definitions of recent screening are consistent with US Preventive Services Task Force (USPSTF) recommendations in effect in 2015. We defined recent breast cancer screening as having received a mammogram within 2 years, recent cervical cancer screening as having a Pap test within 3 years (among women without hysterectomy), and recent colorectal cancer screening as either FOBT within the past year, or flexible sigmoidoscopy within 5 years and FOBT within 3 years, or colonoscopy within 10 years. We used NHIS data from the 2000 through 2015 cycles to describe trends in breast, cervical, and colorectal cancer screening. We examined routine PSA testing in the past year among men aged 50 or older to examine change in use following the 2012 USPSTF guideline revision that recommended against routine screening using PSA (3).

### Statistical analysis

We calculated the proportion of respondents who reported receiving a recent screening test for each type of cancer after adjustment for covariates. We used multivariable logistic regression models to estimate the adjusted association between sociodemographic and access-to-care factors for each type of screening. Independent variables were age, race/ethnicity, education, annual income as a percentage of the federal poverty level, length of US residency, health insurance status and type, having a usual source of care, and consulting a doctor in the past 12 months. Among women, we included consulting a gynecologist in the past 12 months. From logistic regression results, we estimated predictive margins, which are adjusted proportions of screened individuals (6). The predictive margin is computed for a specific group (eg, type of health insurance) as the sample weighted average of the predicted responses from the logistic regression model, assigning each individual in the sample to that group membership while keeping all other covariates unchanged.

Using combined data from all NHIS years, we used multivariable logistic regression models to assess trends over time. Survey year was included as an independent variable, adjusting for the covariates described above. We assessed interactions between survey year and usual source of care or insurance type by including the appropriate product terms in the logistic regression models. We dropped nonsignificant interactions from the final model by using backward elimination (setting 2-sided α = 0.05). We present figures depicting the time trends as predictive margins. Statistical testing was based on the Wald *F* statistic for jointly testing that all regression coefficients for a given covariate were equal to zero. We used 95% confidence intervals (CIs) around the predictive margins to allow for informal pairwise comparisons, although examining overlap between CIs to determine significance is a conservative method. The National Center for Health Statistics imputed missing income data by using multiple imputation. All analyses were performed by using SAS version 9.3 (SAS Institute Inc) and SUDAAN version 11.0.1 (RTI International) to account for the complex sampling design and to allow for sample weighted estimation. All hypothesis tests were considered significant if the 2-sided *P* value unadjusted for multiple comparisons was less than .05.

## Results

**Figure 1:**
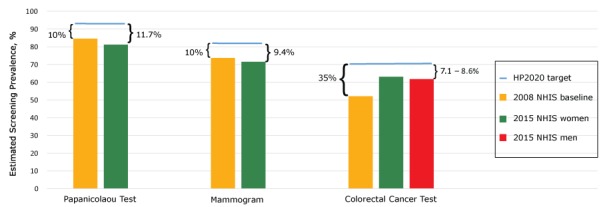
Progress toward meeting Healthy People 2020 cancer screening targets (2), National Health Interview Survey (NHIS) 2008 and 2015 estimates of cancer screening test use based on US Preventive Services Task Force recommendations: Papanicolaou test among women aged 21 to 65 in past 3 years, mammogram among women 50 to 74 within past 2 years, colorectal cancer tests among adults aged 50 to 75 years (fecal occult blood test [FOBT] within past year or flexible sigmoidoscopy past within 5 years and FOBT within past 3 years or colonoscopy within past 10 years). Healthy People 2020 targets represent improvements over 2008 baseline age-adjusted screening levels of 10% for Papanicolaou test, 10% for mammography, and 35% for colorectal cancer testing. Brackets indicate gap between NHIS 2015 reported screening and Healthy People 2020 targets. Abbreviation: NA, not applicable. **Table:** 

In 2015, most women reported having recent screening for cervical, breast, and colorectal cancer (Table 1). However, estimates for all tests, for both women and men, fell short of Healthy People 2020 targets (Figure 1).

**Table 1:** Screening for Cervical and Breast Cancer Among Women, by Demographic Characteristics and Access to Care, National Health Interview Survey, United States, 2015

Characteristic	Cervical Cancer, Papanicolaou Test Within Past 3 years			Breast Cancer, Mammogram Within Past 2 years		
	No.	Predictive Margin^a^ (95% CI)	*P *Value	No.	Predictive Margin^a^ (95% CI)	*P *Value
**Total**	10,431	81.3 (80.3–82.3)	NA	6,663	71.7 (70.2–73.1)	NA
**Age group (y)**						
21–30	2,558	78.5 (76.2–80.6)	<.001	—b	—b	.02
31–40	2,624	84.8 (83.0–86.5)		—b	—b	
41–50	2,172	82.1 (80.1–83.9)		—b	—b	
50–64	NA	NA		4,255	70.5 (68.7–72.3)	
51–65	3,077	80.5 (78.8–82.1)		NA	NA	
65–74	—b	—b		2,408	73.9 (71.5–76.1)	
**Education**						
Less than high school	1,213	78.1 (74.8–81.1)	<.001	855	69.3 (64.9–73.4)	.09
High school graduate	2,138	78.8 (76.7–80.9)		1,687	70.4 (67.6–73.1)	
Some college or associate degree	3,426	81.4 (79.7–83.1)		2,164	70.6 (67.9–73.2)	
College graduate	3,654	84.4 (82.7–86.0)		1,957	75.0 (72.1–77.7)	
**Annual income, percentage of federal poverty level**						
<139%	2,941	80.2 (78.1–82.1)	.06	1,542	67.1 (63.2–70.8)	.003
139%–250%	2,066	80.1 (78.0–82.0)		1,302	67.5 (63.6–71.2)	
251%–400%	1,957	80.3 (78.0–82.4)		1,299	73.9 (70.7–76.8)	
>400%	3,467	83.7 (81.6–85.6)		2,520	74.6 (71.9–77.1)	
**Usual source of health care**						
None or hospital ER	1,403	77.2 (74.4–79.7)	<.001	388	53.3 (45.8–60.7)	<.001
Has usual source	9,028	82.2 (81.1–83.3)		6,275	72.7 (71.2–74.2)	
**Health insurance**						
Private/military	6,997	82.8 (81.5–84.0)	<.001	4,374	74.4 (72.3–76.3)	<.001
Public only^c^	2,124	79.1 (76.6–81.3)		1,922	68.1 (64.7–71.2)	
Uninsured	1,310	78.0 (75.2–80.5)		367	54.4 (47.5–61.2)	
**Race/ethnicity**						
Hispanic	2,089	83.8 (81.4–86.0)	<.001	823	79.3 (74.8–83.2)	<.001
Non-Hispanic white	6,033	80.3 (78.8–81.7)		4,517	70.0 (68.0–71.9)	
Non-Hispanic black	1,544	84.5 (82.4–86.4)		971	77.1 (73.6–80.3)	
Non-Hispanic Asian	649	77.0 (72.7–80.8)		294	65.0 (57.6–71.7)	
Non-Hispanic other^d^	116	77.8 (66.5–86.1)		58	69.7 (50.6–83.8)	
**Length of US residency**						
In United States <10 years	458	71.3 (65.4–76.6)	<.001	69	69.0 (56.3–79.4)	.87
In United States ≥10 years	1,759	81.1 (78.4–83.6)		957	70.9 (65.7–75.6)	
US-born	8,214	81.9 (80.7–83.1)		5,637	71.8 (70.3–73.4)	
**Consulted doctor in past 12 months**						
Yes	7,103	84.1 (83.0–85.2)	<.001	5,411	74.2 (72.5–75.8)	<.001
No	3,328	76.5 (74.6–78.2)		1,252	61.0 (57.7–64.2)	
**Consulted OB/GYN in past 12 months**						
Yes	4,662	96.0 (95.2–96.7)	<.001	1,855	86.6 (84.2–88.7)	<.001
No	5,769	69.9 (68.3–71.5)		4,808	65.1 (63.1–67.1)	

Abbreviations: CI, confidence interval; NA, not applicable.

a Proportions of screened individuals adjusted for all covariates in table. Values are computed for a specific group (eg, type of health insurance) as the sample weighted average of the predicted responses from the logistic regression model, assigning each individual in the sample to that group membership while keeping all other covariates unchanged. The sample totals for this table are crude estimates of the proportion screened in the total population.

b Screening is not recommended for this age group.

c Medicare and/or Medicaid.

d American Indian, Alaska Native, and Pacific Islander.

Screening Test	Prevalence (Predictive Margin) by Year, %			
	2008 NHIS	2015 NHIS Females	2015 NHIS Males	HP2020 Target
Papanicolaou test	84.5	81.3	NA	93.0
Mammogram	73.7	71.7	NA	81.1
Colorectal cancer tests	52.1	63.4	61.9	70.5

### Papanicolaou test

Among women aged 21 to 65, 81.3% (95% CI, 80.3-82.3) reported having a recent Pap test (Table 1). Reporting a recent Pap test was least likely among women aged 21 to 30 (predictive margin [PM], 78.5; 95% CI, 76.2–80.6) or 51 to 65 (PM, 80.5; 95% CI, 78.8–82.1) and among women with less than a high school education (PM, 78.1; 95% CI, 74.8–81.1), women with no usual source of care (PM, 77.2; 95% CI, 74.4–79.7), women who were uninsured (PM, 78.0; 95% CI, 75.2–80.5) or had public insurance only (PM, 79.1, 95% CI, 76.6–81.3), non-Hispanic Asian women (PM, 77.0; 95% CI, 72.7–80.8), and women who were US residents for less than 10 years (PM, 71.3; 95% CI, 65.4–76.6). Women who had not visited a doctor (PM, 76.5; 95% CI 74.6–78.2) or obstetrician/gynecologist (OB/GYN) (PM, 69.9; 95% CI, 68.3–71.5) in the past 12 months were least likely to report a recent Pap test.

### Mammography

Among women aged 50 to 74, 71.7% (95% CI, 70.2–73.1) reported a recent mammogram (Table 1). Reporting a recent mammogram was least likely among women aged 50 to 64 (PM, 70.5; 95% CI, 68.7–72.3), women with annual incomes less than 139% of the federal poverty level (PM, 67.1%; 95% CI, 63.2–70.8), women without a usual source of care (PM, 53.3; 95% CI, 45.8–60.7), uninsured women (PM, 54.4; 95% CI, 47.5–61.2) or publicly insured (PM, 68.1; 95% CI, 64.7–71.2), and non-Hispanic Asian women (PM, 65.0; 95% CI, 57.6–71.7) and non-Hispanic white women (PM, 70.0; 95% CI, 68.0–71.9). Women without recent consults with a doctor (PM, 61.0; 95% CI, 57.7–64.2) or OB/GYN (PM, 65.1; 95% CI, 63.1–67.1) were also less likely to report a recent mammogram.

### Colorectal cancer screening

Among women aged 50 to 75, 63.4% (95% CI, 61.7-65.0) reported a recent colorectal cancer screening test (Table 2). Reporting a recent screening test was least likely among women aged 50 to 64 (PM, 60.6; 95% CI, 58.3–62.8), women with less than a high school education (PM, 58.2; 95% CI, 53.6–62.7), women with an annual income less than 139% of the federal poverty level (PM, 55.3; 95% CI, 51.2–59.2), women without a usual source of health care (PM, 45.9, 95% CI, 38.9–53.1), uninsured women (PM, 45.4; 95% CI, 37.9–53.2), non-Hispanic Asian women (PM, 54.7; 95% CI, 47.1–62.1), and women who had not consulted a doctor in the past year (PM, 49.1; 95% CI, 45.2–53.1) or an OB/GYN in the past year (PM, 60.7; 95% CI, 58.7–62.5).

**Table 2:** Screening^a^ for Colorectal Cancer Among Women, by Demographic Characteristics and Access to Care, National Health Interview Survey, United States, 2015

Characteristic	No.	Predictive Margin^b ^(95% CI)	*P *Value
**Total**	6,816	63.4 (61.7–65.0)	NA
**Age group, y**			
50–64	4,232	60.6 (58.3–62.8)	<.001
65–75	2,584	68.8 (66.3–71.2)	
**Education**			
Less than high school	885	58.2 (53.6–62.7)	.03
High school graduate	1,748	61.5 (58.5–64.5)	
Some college or associate degree	2,204	64.4 (61.6–67.1)	
College graduate	1,979	65.8 (62.6–68.9)	
**Annual income, percentage of poverty level**			
<139	1,575	55.3 (51.2–59.2)	<.001
139–250	1,350	59.6 (55.3–63.7)	
251–400	1,340	64.0 (60.5–67.4)	
>400	2,551	67.9 (65.2–70.4)	
**Usual source of health care**			
None or hospital emergency department	394	45.9 (38.9–53.1)	<.001
Has usual source	6,422	64.2 (62.5–65.9)	
**Health insurance**			
Private/military	4,455	64.2 (62.2–66.2)	<.001
Public only^c^	1,990	64.0 (60.5–67.4)	
Uninsured	371	45.4 (37.9–53.2)	
**Race/ethnicity**			
Hispanic	835	59.3 (54.1–64.2)	.05
Non-Hispanic white	4,631	64.5 (62.4–66.5)	
Non-Hispanic black	989	64.8 (60.9–68.5)	
Non-Hispanic Asian	303	54.7 (47.1–62.1)	
Non-Hispanic other^d^	58	50.3 (30.1–70.4)	
**Length of US residency**			
<10 years	73	55.1 (38.8–70.4)	.41
≥10 years	980	61.3 (56.3–66.1)	
US-born	5,763	63.8 (62.0–65.6)	
**Consulted doctor in past 12 months**			
Yes	5,542	66.5 (64.7–68.2)	<.001
No	1,274	49.1 (45.2–53.1)	
**Consulted OB/GYN in past 12 months**			
Yes	1,872	69.2 (66.3–72.0)	<.001
No	4,944	60.7 (58.7–62.5)	

Abbreviations: CI, confidence interval; OB/GYN, obstetrician/gynecologist’ NA, not applicable.

a Fecal occult blood test (FOBT) in the past year, flexible sigmoidoscopy in past 5 years with FOBT in past 3 years, or colonoscopy in past 10 years.

b Proportions of screened individuals adjusted for all covariates in table. Values are computed for a specific group (eg, type of health insurance) as the sample weighted average of the predicted responses from the logistic regression model, assigning each individual in the sample to that group membership while keeping all other covariates unchanged. The sample totals for this table are crude estimates of the proportion screened in the total population.

c Medicare and/or Medicaid.

d American Indian, Alaska Native, and Pacific Islander.

Among men aged 50 to 75, 61.9% (95% CI, 60.0–63.7) reported having recent colorectal cancer screening (Table 3). Reporting recent screening was lowest among men aged 50 to 64 (PM, 57.4; 95% CI, 55.0–59.7), men with less than a high school education (PM, 53.9; 95% CI, 48.4–59.3), men with an annual income less than 139% of the federal poverty level (PM, 55.2; 95% CI, 50.7–59.7), men without a usual source of health care (PM, 42.2; 95% CI, 35.5–49.1), uninsured men (PM, 49.0; 95% CI, 40.8–57.2), and men who had not consulted a doctor in the past 12 months (PM, 47.8; 95% CI, 44.2–51.5).

**Table 3:** Cancer Screening Among Men, by Demographic Characteristics and Access to Care, National Health Interview Survey, United States, 2015

Characteristic	Colorectal Cancer, Recent Test^a^			Prostate Cancer, PSA Test in Past Year		
	No.	Predictive Margin (95% CI)	*P* Value	No.	Predictive Margin^b^ (95% CI)	*P* Value
**Total**	5,679	61.9 (60.0–63.7)	NA	6,636	35.8 (34.2–37.4)	NA
**Age group, y**						
50–64	3,606	57.4 (55.0–59.7)	<.001	3,513	30.3 (28.3–32.4)	<.001
65–74	NA	NA		1,903	44.7 (41.8–47.6)	
65–75	2,073	71.6 (69.3–73.9)		NA	NA	
>75	—a	—a	—a	1,220	40.6 (36.8–44.4)	
**Education**						
Less than high school	770	53.9 (48.4–59.3)	<.001	1,013	26.9 (22.8–31.3)	<.001
High school graduate	1,504	58.5 (54.7–62.1)		1,786	32.7 (29.7–35.9)	
Some college or associate degree	1,658	60.1 (56.9–63.1)		1,829	34.4 (31.5–37.5)	
College graduate	1,747	69.0 (65.8–72.0)		2,008	42.3 (39.1–45.6)	
**Annual income, percentage of federal poverty level**						
<139%	1,076	55.2 (50.7–59.7)	.01	1,213	30.8 (26.6–35.4)	<.001
139%–250%	1,051	59.9 (55.5–64.1)		1,325	32.7 (29.0–36.7)	
251%–400%	1,091	61.0 (56.8–65.0)		1,365	32.2 (28.9–35.7)	
>400%	2,461	64.9 (62.0–67.6)		2,733	39.5 (37.1–42.0)	
**Usual source of health care**						
None or hospital emergency department	589	42.2 (35.5–49.1)	<.001	619	20.2 (13.7–28.6)	<.001
Has usual source	5,090	63.6 (61.7–65.6)		6,017	36.6 (34.9–38.4)	
**Health insurance**						
Private or military	3,815	63.6 (61.4–65.8)	.003	4,387	37.3 (35.3–39.3)	.01
Public only^c^	1,456	59.4 (55.4–63.3)		1,844	32.7 (29.6–36.0)	
Uninsured	408	49.0 (40.8–57.2)		405	26.0 (17.8–36.2)	
**Race/ethnicity**						
Hispanic	624	57.7 (51.3–63.8)	.12	713	34.3 (29.0–40.1)	<.001
Non-Hispanic white	4,034	62.6 (60.3–64.8)		4,786	36.9 (35.0–38.8)	
Non-Hispanic black	716	65.5 (61.3–69.4)		790	38.3 (34.1–42.7)	
Non-Hispanic Asian	251	51.1 (40.9–61.2)		289	16.8 (11.6–23.8)	
Non-Hispanic other^d^	54	62.9 (47.4–76.1)		58	29.1 (13.9–50.9)	
**Length of US residency**						
<10 years	51	52.1 (38.4–65.5)	.33	51	29.9 (17.0–47.2)	.54
≥10 years	783	60.4 (54.8–65.6)		907	37.8 (32.4–43.5)	
US-born	4,845	62.2 (60.1–64.3)		5,678	35.6 (33.8–37.4)	
**Consulted a doctor in past 12 months**						
Yes	4,338	65.8 (63.6–67.9)	<.001	5,163	39.6 (37.8–41.4)	<.001
No	1,341	47.8 (44.2–51.5)		1,473	18.3 (15.3–21.8)	

Abbreviations: CI, confidence interval, NA, not applicable.

a Fecal occult blood test (FOBT) in the past year, flexible sigmoidoscopy in past 5 years with FOBT in past 3 years, or colonoscopy in past 10 years. Colorectal cancer screening is not recommended for people older than 75.

b Proportions of screened individuals adjusted for all covariates in table. Values are computed for a specific group (eg, type of health insurance) as the sample weighted average of the predicted responses from the logistic regression model, assigning each individual in the sample to that group membership while keeping all other covariates unchanged. The sample totals for this table are crude estimates of the proportion screened in the total population.

c Medicare and/or Medicaid.

d American Indian, Alaska Native, and Pacific Islander.

### Prostate-specific antigen test

Among men aged 50 years or older, 35.8% (95% CI, 34.2–37.4) reported having a PSA test in the past year (Table 3). Reporting a recent PSA test was lowest among men aged 50 to 64 years (PM, 30.3; 95% CI, 28.3–32.4), men with less than a high school education (PM, 26.9; 95% CI, 22.8–31.3), men with annual income less than 139% of the federal poverty level (PM, 30.8; 95% CI, 26.6–35.4), men with no usual source of health care (PM, 20.2; 95% CI, 13.7–28.6), uninsured men (PM, 26.0; 95% CI, 17.8–36.2), non-Hispanic Asian men (PM, 16.8; 95% CI, 11.6–23.8), and men who had not consulted a doctor in the past 12 months (PM, 18.3; 95% CI, 15.3–21.8).

### Temporal trends in use of cancer screening tests, 2000–2015

**Figure 2:**
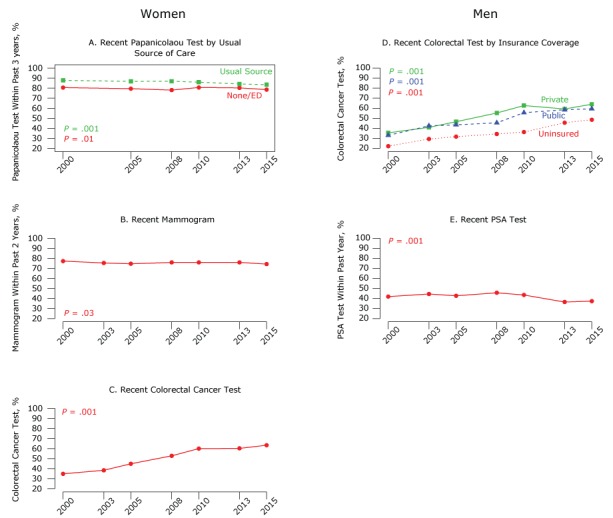
Trends in use of cancer screening tests among women and men, National Health Interview Survey, 2000–2015 (www.cdc.gov/nchs/nhis/index.htm). A. Prevalence of having a Papanicolaou test within past 3 years among women aged 20 to 65. B. Prevalence of having a mammogram among women aged 50 to 74 within past 2 years. C. Prevalence of having a recent colorectal cancer test among women aged 50 to 75 (for colorectal cancer tests, recent is defined as having a fecal occult blood test [FOBT] in the past year, flexible sigmoidoscopy in past 5 years with FOBT in past 3 years, or colonoscopy in past 10 years). D. Prevalence of having a recent colorectal cancer test, by insurance coverage, among men aged 50 to 75. E. Prevalence of having a PSA test among men aged 50 and older. Estimates were adjusted for age, education, poverty, usual source of care, type of health insurance, race/ethnicity, length of US residency, physician visit in the past year, and among women, OB/GYN visit in the past year. Abbreviations: ED, emergency department; PSA, prostate-specific antigen.

We assessed trends in recent cancer screening test use based on data from the 2000, 2003, 2005, 2008, 2010, 2013, and 2015 NHIS cycles (Figure 2).

**Table:** 

Papanicolaou Test, Year	**Access to Health Care**	**%**
2000	None/emergency department	80.5
2005	None/emergency department	79.4
2008	None/emergency department	78.0
2010	None/emergency department	80.6
2013	None/emergency department	80.2
2015	None/emergency department	78.5
2000	Usual source	87.7
2005	Usual source	86.7
2008	Usual source	86.8
2010	Usual source	85.9
2013	Usual source	84.1
2015	Usual source	83.4

**Table:** 

Mammogram, Year	%
2000	77.2
2003	75.3
2005	74.5
2008	75.8
2010	75.9
2013	75.9
2015	74.2

**Table:** 

**Colorectal Cancer, Women, Year**	**%**
2000	34.8
2003	38.3
2005	44.8
2008	52.7
2010	59.9
2013	60.1
2015	63.3

**Table:** 

Colorectal Cancer, Men, Year	Insurance	%
2000	Private	35.4
2003	Private	40.9
2005	Private	46.5
20008	Private	55.2
2010	Private	62.6
2013	Private	59.2
2015	Private	63.9
2000	Public	32.9
2003	Public	42.4
2005	Public	43.4
2008	Public	45.4
2010	Public	55.5
2013	Public	58.4
2015	Public	59.4
2000	Uninsured	22.2
2003	Uninsured	29.3
2005	Uninsured	31.6
2008	Uninsured	34.3
2010	Uninsured	36.2
2013	Uninsured	45.5
2015	Uninsured	48.5

**Table:** 

PSA Test, Year	%
2000	41.8
2003	44.4
2005	42.6
2008	45.6
2010	43.4
2013	36.4
2015	37.3

Pap test use declined significantly by 4.3% from 2000 to 2015 among women with a usual source of care (Figure 2A). No significant change in use was seen among women with no usual source of care. Adjusted mammography rates declined significantly by 3.0% from 2000 to 2015, although rates remained relatively high throughout the 2000–2015 period (Figure 2B). No interactions between survey year and usual source of care or insurance type were significant for mammography use. Colorectal cancer screening rates among women increased significantly from 2000 to 2010, by 25.1%. The rate remained stable from 2010 to 2013, then increased slightly in 2015 for an overall increase of 28.5% (Figure 2C). No interactions between sociodemographic characteristics, access to care, and recent colorectal cancer tests were significant among women.

Among men, colorectal cancer screening use increased significantly for all men and over time among all insurance groups, but varied by insurance type (Figure 2, D). From 2000 through 2010, the increase was larger among men with private insurance (27.2%) than other insurance groups. The rate then declined in 2013 before increasing again in 2015 for an overall increase of 28.5%. The increases among men with public insurance (26.5%) and among the uninsured (26.3%) were more linear during the period. Use of an annual PSA test declined significantly (9.2%) from 2008 through 2013 but remained stable from 2013 through 2015, dropping 4.5% overall (Figure 2E). No significant interactions between sociodemographic characteristics or access to care and having a recent PSA test were observed.

## Discussion

The 2015 NHIS findings show that use of cancer screening tests for cervical, breast, and colorectal cancer remained below Healthy People 2020 targets. Significant variation in test use by sociodemographic characteristics and access to care factors remained after adjustment. For all tests studied, the lowest screening rates were generally associated with having no usual source of care, no insurance, not having seen a physician in the past 12 months, and identifying as non-Hispanic Asian. Younger age, lower income, and fewer years of education were also consistently associated with lower prevalence of screening for both men and women. Examination of trends adjusted for these factors showed a persistent screening disparity over time for those uninsured or with no usual source of care. Patterns of disparities in screening test use found among population subgroups in 2015 were consistent with those found in previous cycles of NHIS (7–9). A striking exception was that non-Hispanic white women were less likely than non-Hispanic black women or Hispanic women to obtain Pap tests or mammograms in 2015.

Only use of colorectal cancer screening increased in 2015 (10). Although rates improved for most groups examined, screening for colorectal cancer was lowest among uninsured women and men (women, 45%; men, 49%), those with no usual source of care (women, 46%; men, 42%), those with no doctor consult in the past 12 months (women, 49%; men, 48%), and non-Hispanic Asians (women, 55%; men, 51%). Colorectal cancer screening is effective and can prevent cancer; however, even in 2015, when rates for Pap tests and mammography were 80% and 70%, respectively, rates for colorectal cancer screening were just above 60%. Focused public health efforts to promote colorectal cancer screening may have helped increase rates over the past 10 years. However, sustained efforts will be necessary to increase awareness of the need for screening, continued expansion of insurance coverage, and use of electronic medical records with automatic reminders to patients and physicians (11) for further increases in test use, particularly among those subgroups with the lowest use.

Pap test and mammography rates showed slight declines in use from 2000 to 2015, and overall measures remained below national targets, especially for specific subgroups of women. Though Pap test screening exceeded 80%, and mammography exceeded 70%, test rates were lower than the Healthy People 2020 targets of 93% for Pap tests and 81% for mammography (2,9). In 2017, Watson and colleagues estimated that in 2015, 14 million women aged 21 to 65 had not had a Pap test in the past 3 years (12). To reverse the downward trend and improve outreach to those rarely or never screened, educational and promotional interventions or information dissemination efforts will be needed, and accurate monitoring will be important. Enhanced efforts are needed to increase screening among underserved groups.

The prevalence of use of the PSA test dropped 5 percentage points, from 42.6% in 2005 to 37.3% in 2015, extending earlier trends documenting reduction in test use. In 2012, USPSTF recommended against routine PSA testing and since there has been a drop in the test being offered by physicians and used by patients (13,14) consistent with our findings. In 2015, Drazer and colleagues reported the largest decreases were observed among white men and men aged 50 and older (13); however, screening still occurred in 36% of men. Approximately one-third of men older than 75 with a life expectancy of less than 10 years were screened in 2013, although screening is never recommended for such patients. A 2017 update to the 2012 USPSTF PSA screening recommendation (15) recommends that men aged 55 to 69 years discuss the potential benefits and harms of PSA screening with their clinician and incorporate their values and preferences in the screening decision. The 2017 update also recommends men aged 70 and older should not be screened, and men at higher risk of prostate cancer, such as African American men or those with a family history, should consult their clinician about the appropriateness of considering screening before age 55. Therefore, it will be important to continue to monitor test use to determine if prevalence of testing rises following the 2017 update.

The cancer screening objectives for Healthy People 2020 provide targets for monitoring cancer incidence, mortality, and survival. Our analysis of the 2015 NHIS showed that halfway through the Healthy People 2020 decade, estimates for use of cervical, breast, and colorectal cancer screening tests are well below Healthy People 2020 targets. An estimated 24.4 million people would need to be screened in the United States to achieve the National Colorectal Cancer Roundtable target of screening 80% of age-appropriate individuals by 2018 (16), including 3.9 million people expected to reach screening age from 2015 through 2018.

One approach to improving screening use across all subgroups would be for physicians to recommend screening to all age-appropriate patients (17), including traditionally underserved groups (low-income, uninsured, low acculturation or assimilation, Hispanic, and non-Hispanic Asian) (18,19). Although having insurance coverage does not ensure doctor visits, universal health insurance coverage and universal access to a usual source of health care would likely increase physician access to all groups to encourage screening. Although it would be optimal for physicians to recommend screening to less acculturated and uninsured Hispanics and Asians and in their own languages (20,21), studies suggest that physician enthusiasm and outreach with tailored or innovative strategies to educate and inform (22) may increase knowledge and intention to screen among underserved groups, such as Hispanics, Asians, the uninsured, and the less educated. Culturally tailored strategies may be particularly effective for Asians disproportionately affected by discordance in patient–provider language and gender (provider and patient being of different genders) (22,23). More research and evaluation of public health campaigns designed to increase screening among underserved groups are needed.

Evidence-based, multicomponent interventions have potential to substantially increase screening rates among population groups with low screening rates (24,25). Efforts that result in better continuity and coordination of care, such as community-based patient navigation programs, may be particularly useful. Care coordination is a key strategy with potential to improve health care system effectiveness, safety, and efficiency. Well-designed, targeted coordination efforts can improve patient, provider, and payer outcomes (26). The Community Preventive Services Task Force findings suggest that multicomponent interventions that increase community demand and access along with increasing provider delivery of services show the greatest screening effects (27). Combined with provision of appropriate follow-up care and treatment, these interventions may improve health for the underserved.

Our study had limitations. Survey data were self-reported and not confirmed by medical record review. Data did not distinguish diagnostic tests from screening and may have overestimated test use for screening purposes. In addition, American Indian, Alaska Native, and Pacific Islander samples were too small to analyze separately and were included in a “non-Hispanic other” group; limiting our knowledge about these subgroups. Monitoring screening among all these groups is essential to determine what allocation of resources and efforts is necessary to increase their screening participation. Lastly, beginning in 2010, NHIS asked separate questions for each type of endoscopy (sigmoidoscopy vs colonoscopy). Before 2010, respondents were asked which endoscopy type was the most recent test. If the most recent test was a sigmoidoscopy that occurred within 10 years but did not meet recommendations, we assumed that the sigmoidoscopy was not preceded by a colonoscopy within 10 years, and that screening was not up to date. Although this may underestimate screening slightly, having a sigmoidoscopy following a recent colonoscopy is likely to be rare.

Given the rate of change between 2000 and 2010, accelerated uptake is needed to reach Healthy People 2020 targets for screening for cervical, breast, and colorectal cancers. Again in 2015, the uninsured and those with no usual source of care were least likely to have received a screening test within recommended timelines. Continued efforts are needed to reduce structural barriers for access to medical care and to increase physician contact to increase the proportion of people counseled and participating in cancer screening, particularly among underserved subgroups. In addition to more consistent physician recommendation, screening promotion efforts for all tests directed at patients need to identify and address barriers among Asians, those without a usual source of care, and the uninsured. Culturally and linguistically appropriate and targeted interventions have been shown to maintain high rates of Pap test and mammography screening and to continue to increase colorectal cancer screening (28–30). Appropriate diagnosis, timely follow-up, and effective treatment will help to make inroads toward reducing overall cancer burden and improving health equity in cancer outcomes for all. Continued monitoring is critical to learn how screening rates compare with Healthy People 2020 targets, and it is important to adjust for sociodemographic factors because of changes in insurance coverage and racial/ethnic composition in the population over time.

## Post-Test Information

To obtain credit, you should first read the journal article. After reading the article, you should be able to answer the following, related, multiple-choice questions. To complete the questions (with a minimum 75% passing score) and earn continuing medical education (CME) credit, please go to http://www.medscape.org/journal/pcd. Credit cannot be obtained for tests completed on paper, although you may use the worksheet below to keep a record of your answers. You must be a registered user on Medscape.org. If you are not registered on Medscape.org, please click on the "Register" link on the right hand side of the website to register. Only one answer is correct for each question. Once you successfully answer all post-test questions you will be able to view and/or print your certificate. For questions regarding the content of this activity, contact the accredited provider, CME@medscape.net. For technical assistance, contact CME@webmd.net. American Medical Association’s Physician’s Recognition Award (AMA PRA) credits are accepted in the US as evidence of participation in CME activities. For further information on this award, please refer to http://www.ama-assn.org/ama/pub/about-ama/awards/ama-physicians-recognition-award.page. The AMA has determined that physicians not licensed in the US who participate in this CME activity are eligible for ***AMA PRA Category 1 Credits™***. Through agreements that the AMA has made with agencies in some countries, AMA PRA credit may be acceptable as evidence of participation in CME activities. If you are not licensed in the US, please complete the questions online, print the AMA PRA CME credit certificate and present it to your national medical association for review.

## Post-Test Questions

### Study Title: Patterns and Trends in Cancer Screening in the United States

#### CME Questions

1. You are advising a large health maintenance organization regarding increasing uptake of appropriate cancer screening. On the basis of the analysis of 2000 to 2015 National Health Interview Survey (NHIS) data by Hall and colleagues, which one of the following statements about specific trends in prevalence of cancer screening is correct?
  - Papanicolaou (Pap) test and mammography rates increased slightly from 2000 to 2015
  - Pap screening exceeded 80% and mammography 70%, but test rates were lower than the Healthy People 2020 (HP2020) targets of 93% and 81%, respectively
  - PSA screening increased slightly from 2005 to 2015
  - In 2015, colorectal cancer (CRC) screening met the HP2020 target
    study period, but similar to cervical and breast cancer screening, it still remained below HP2020 targets.
2. According to the analysis of 2000 to 2015 NHIS data by Hall and colleagues, which one of the following statements about factors associated with underuse of cancer screening is correct?
  - Factors associated with lower screening rates were lack of physician contact in the last year, no usual source of care, and lack of insurance coverage
  - After adjustment for sociodemographics and access to care factors, there were no significant disparities in test use
  - In terms of racial groups, the lowest screening rates were in non-Hispanic blacks
  - In 2015, non-Hispanic white women were more likely than non-Hispanic black women or Hispanic women to have Pap tests or mammograms
3. On the basis of the analysis of 2000 to 2015 NHIS data by Hall and colleagues, which one of the following statements about clinical implications of trends in prevalence of cancer screening is correct?
  - The study was likely to have underestimated test use for screening purposes
  - The study proved lower screening rates in American Indians
  - The only strategy likely to increase screening among Hispanics and Asians is to provide recommendations in their own languages

**Table:** Evaluation

**1. The activity supported the learning objectives.**				
**Strongly Disagree**				**Strongly Agree**
**1**	**2**	**3**	**4**	**5**
**2. The material was organized clearly for learning to occur.**				
**Strongly Disagree**				**Strongly Agree**
**1**	**2**	**3**	**4**	**5**
**3. The content learned from this activity will impact my practice.**				
**Strongly Disagree**				**Strongly Agree**
**1**	**2**	**3**	**4**	**5**
**4. The activity was presented objectively and free of commercial bias.**				
**Strongly Disagree**				**Strongly Agree**
**1**	**2**	**3**	**4**	**5**
